# Does a Mind Need a Body?

**DOI:** 10.1017/S0963180121000049

**Published:** 2021-10

**Authors:** Alex McKeown, David R. Lawrence

**Affiliations:** 1 Department of Psychiatry, Wellcome Centre for Ethics and Humanities, University of Oxford, Oxford, UK; 2 Centre for Ethics and Law in the Life Sciences, Durham Law School, Durham University, Durham, UK

## Alex McKeown: Opening Argument in the Affirmative

This question of whether the mind needs a body is a long-standing philosophical dispute, so I do not imagine that we are going to settle it once and for all today; nevertheless, I am going to argue that a mind does, in fact, need a body.

Some of these remarks are reflected in my paper “What Do We Owe to Novel Synthetic Being and How Can We Be Sure?” which will be appearing in the July issue of the Cambridge Quarterly. In that paper, I argue that any account of our obligations to Novel Synthetic Beings (NSBs), whether they are machine-based or (synthetic) biologically based, will be incomplete and faulty if we only consider them in terms of how “intelligent” they are, in the narrow cognitive sense that we tend to often use the word. It is easy to overlook the role of embodiment and assume that the only component that is essential for moral status is mental sophistication, since it is this that enables a being to self-reflect, have a conception of its own future, plans, values, an awareness of its own desires, and so on. But in fact, as I argue in the paper, doing this excludes the role that embodiment has to play in the having of those capacities.

In the paper, I defend the view that we will have an incomplete account of what we owe to NSBs, what their rights are, and what they are entitled to, if we do not take into account the terms of their embodiment, what it enables and forbids them to *do.* My argument is that the physical aspect of their existence cannot be disaggregated from their mental capacities, and so to properly understand the nature of minds, in artificially intelligent beings in the case of the paper, we must also take into account how the putatively exclusively mental processes are physically instantiated. Those thoughts are the basis for where we stand now. I am arguing for the purposes of this debate that a mind does indeed need a body.

As a starting point, I think there are two interpretations of “need” at play here. The first is whether or not the processes of mind are *intelligible* in the absence of the body; that is to say, whether we can even *conceive of them as occurring* in the absence of the body, or whether we can get a sense of what the mental is without taking into account the physical vehicle in which it is instantiated.

The second interpretation of “need” is not just about intelligibility but whether, as a matter of *logic*, there *must* be body for there to be mind. Since, for reasons I will defend, the answer to this second interpretation is “yes,” insofar as mind cannot logically exist without body, the other question, of intelligibility, is a red herring. This is because if we hold that the mental logically requires the physical, then the notion of disembodied mind cannot be properly intelligible.

My claim that there is no mind without body is grounded on an underlying ontological physicalist naturalism, and more specifically Strawsonian physicalist naturalism.^1^ It is fair to say that if you dispute the coherence of the underlying ontological picture, you will find the arguments that I make unpersuasive, and I flag this as a caveat and potential weakness of the arguments that I am going to make. Nevertheless, I find it a persuasive ontological account, and according to this account, the physical is coextensive with all there is. Everything exists within the physical universe. There is nothing beyond it; the substrate of everything is physical. As such, *all* phenomena are grounded in the physical. Because nothing exists beyond the physical, anything and everything that exists is necessarily physically instantiated, including processes of mind. Even though the association between the two can seem mysterious, nevertheless, there are no processes in mind that are not physically instantiated.

There is a second and more difficult challenge to my argument that I also need to flag. This is the difference between embodiment and *mere* instantiation. I accept this challenge. All bodies are physical, but not everything physical is going to count as body—or at least there can be legitimate dispute about what a body is. For example, it does not appear to make sense to say that the air could count as “a body,” just because it is physical.

In response to this challenge, however, one could say that equally we do not have the privilege of defining what definitely is and *is not* a body. We know that the bodies are the delimited seats of conscious experience in humans and animals, but they do not necessarily have to rigidly conform to the way that we use the word “body” currently in perpetuity. And if our technological trajectory is one where at some point we will be able to create novel forms of consciousness—true AI, for example—then at some point, questions about the meaning and self-understanding of their physical form are likely to arise.

If both the development of AI and synthetic biology were to succeed in this way in future, then we may be on the path to widening the scope of what count as bodies. We already know there is more than one kind of body: human bodies vary, within certain limits, and there are numerous kinds of animal bodies. Therefore, the scope is wide for what other physical forms could legitimately count as bodies. A useful intuition pump here for starting to think about this is Chrisley’s fourfold typology of embodiment, which has been developed in the context of AI but is valuable for thinking more generally about different ways that embodiment might be legitimately understood.^2^ In addition, what it is to have a mind is integrated with what it is like to exist and experience the world. And the only way to experience the world is by the physical operators that enable you to do that. So, the notion of mind breaks down and becomes unintelligible without that physical coupling being part of the conceptual picture.^3^

This is not true only for relatively cognitively sophisticated beings such as humans, which not only *have* mental processes but are also consciously *aware* of having them. It is easy to forget that what we count as “mental” properties are not necessarily *only* a set of higher-order processes that enable us to have conversations like the one we are having in this debate, but also much more primitive, overtly “physical”—that is to say, rather than overtly intellectual—processes.^4^ We often tend to think of the mental in terms of the former—like the ability to reflect on one’s values, or doing mathematics, or whatever it might be. But of course, even basic functions necessary for getting by in the most simple way^5^—such as the ability to perceive and be aware of one’s environment or negotiate one’s environment via a particular form of locomotion—require both the capacity (realized in a brain or comparable organ or component) to interpret the information coming in *and* the mediation of this information by physical sensing apparatus.^6^ Indeed, given the underlying ontological position from which I am starting, going down to an even lower level of sophistication, mental processes are *necessarily* physically instantiated, or embodied, and as such, the idea of mind existing without an interface with the world outside is incoherent.

So, to summarize briefly: since mind is mediated by the physical, so there are no minds which are not grounded in the physical, and since bodies are necessarily physical, a mind requires a body. A reasonable objection to this final claim is that not everything physical is a body, but the response to that is, again, to say that the notion of a nonphysical body is in coherent, so whatever else a body is or is not, it is definitely *physical.* Therefore, I think what is *really* up for grabs and at the core of the dispute here is what does and does not count as a body.

## David Lawrence: Opening Argument in the Negative

I have a difficult task, I admit, in making a case for the negative. Somewhat controversially I will begin by telling you first why I doubt my own position, and why I had a hard time constructing this stance. You will all be familiar, if not previously so then certainly following Alex’s well-made argument, with the fundamentals of the “mind–body problem” discourse- (physicalist) Monism versus Dualism. An impasse between those who claim that the mind, the self, perhaps consciousness, are intrinsic to the brain and its biology^7^; and those who cannot accept that, who see the mind as something other, is that there is some distinction between mind and matter.^8^ In this debate, it might be more useful to talk about monism in terms of the physical, Alex being so concerned with the material reality of the brain as being all that there is, and so I will use both physicalism and materialism somewhat interchangeably from now on, the distinction between the two not seeming to matter too much for our question.^9^

An easy approach to this argument would have been to decry a purely physicalist approach, to just wholeheartedly endorse dualism, but I do not see it as my place to try to convince you of some ephemeral, invisible other. I would struggle with that position, and I always considered myself a materialist—I struggle to accept arguments for which, by their nature, we cannot present observable evidence. Ironically, I have faith in science explaining everything, so I cannot blindly endorse a classic dualism. Unfortunately for me, that faith, or the desire for that to be true, at least, does not prevent a niggling doubt I have, which I want to explore here.

The question “does a mind need a body?” presupposes a couple of things. It requires us to know what a mind is, and it requires us to know what a body is. The formulation of the question implies, perhaps, that a body is a host for a mind, a place it resides in some way. That works very nicely for my opponent’s physicalist viewpoint—if “mind” is just a word we use for whatever biological processes we do not yet entirely understand (and lest we forget, cannot yet identify)—then those processes need meat, and they need anatomy in which to occur. A body, of course, provides that. We tend to think of a *human* body when we say the word “body,” but since there is not really a platonic idea of what a body is or should be, we know at least that it does not have to be a plantigrade biped with cranium uppermost, like us. Format does not matter much, and is in many ways banal—so long as there is a material structure to support the processes that we tend to call “mind.” Whatever those might prove to be, the body for this view could just be a host for these processes.

But of course, we do not actually tend to have such an open perspective, particularly in general conversation. When we talk about the body as a physical object in this discussion on the mind–body problem, we tend to be actually using it as what Peter Wolfendale refers to as an “index of authenticity,” a “stand-in for whatever it is that supposedly enables actual human cognition…[with] little reason offered for this beyond its centrality to the current form of life we share.”^10^ In other words, we are only really using the term, because it is what we recognize, and not because we actually mean something specific by it. Alex, really, has kindly made this argument for me, much better than I could. “Real meat” is, as Wolfenden puts it, our common denominator, so that is what we tend to think of. To me, that tendency makes us miss something important in this debate, which is that we do not really have any good reason to pick some specific thing as representing “body” here. It also leads us to certain assumptions about what a mind is.

Of course, we can observe some cognitive processes, seeing where and how they take place and connect within our neuroanatomy.^11^ We can observe similar processes in other carbon-based, organic bodies. We can see how those processes might differ slightly in different animals, in different body plans, and in different body complexities. We sometimes use what we understand about one kind of body to deduce the biological processes in another kind of body. We use animal testing for a reason, after all. And I accept some examples of bodies have more complex architecture, more capable hardware, so to speak. They can perform mental processes that others cannot. Clearly, some examples of bodies have the capacity for higher levels of cognition than others. I would not stand here and suggest to you that, in that respect, the body of a mouse is equal to that of an orangutan. But… they are all “A Body.” I will come back to this point.

## The Subjective Mind

The other issue raised by the debate question was that we are assumed to know what a “mind” actually is. As I have said, I find it hard to accept the idea of some nebulous “other” substance interposed on or occupying the same physical space as our body. Most sensations we have are rooted in some observable physical way in our biology. I do not presume to dispute that, but here is the thing I mentioned that creates doubt within me. There is a lot about our physical bodies that might well be physically instantiated, but which we do not experience in any meaningful way. There is nothing much you can describe about the workings of your kidneys, your gall bladder, or your liver—they all fulfil vital, important functions, but they only time we become aware of them is because some malady causes other bodily systems to draw our attention to them. The flipside of all this, of course, is that there is a lot of experience that we *do* perceive, but that is not obviously physically instantiated. Relevantly here, the experiences of our mind are, or at least include, subjective experiences. These would be by nature unobservable, at least in terms of their character.

According to Thomas Nagel, who I accept is very commonly invoked in this debate, this subjectivity defeats reduction, because the subjective character of experience cannot be explained by a system of functional or intentional states.^12^ How can every possible subjective experience—and degree of such—be tied to an individual physical property? It simply cannot be proved, because it is impossible to have an objective answer. Subjective experiences cannot be objective, as they cannot be verified—by nature, they are one point of view. We could not know what it is like for a bat to be a bat, because we can never be a bat ourselves—only imagine it, as a human and from our experience as humans. It is not possible for us to shed that experiential bias.

Daniel Dennett’s argument against Nagel is that the “interesting or theoretically important”^13^ elements of mind or consciousness *could* be observed, and that subjective experience, therefore, does not matter—because the mindset of a bat does not matter. In the grand scheme of things, I suppose this is true, bats are not important, but this seems to me to willfully miss something valuable. A copy of a mind—if such a thing is possible—would not be a copy if it was missing some element. Without the subjective experience, a copy would be a fundamentally impoverished account of that individual and specific mind. In the best case, it would be a blank slate—a mind in function and form, perhaps, but crucially not the one you tried to copy. This is sometimes called a *philosophical zombie.* ^14^

Similarly to the bat, it is not possible for me to know what it is like to be Alex. I cannot know his experience, or at least how he perceives experience. I cannot know how he perceives color—maybe it is the same way as I do, but maybe not, and we just have no way to prove it one way or the other. Even if we were to scan our cognitive activity, and the records seemed comparable, nothing much could arise from that save to demonstrate that our substrate is the same and we operate according to the same laws of physics and biochemistry (which would be some relief, even if not useful). It could not say anything significant about what he experiences, or if our experiences are the same. You cannot reduce things to a pure physicalist view without ignoring that subjectivity.

We have developed various ways of trying to discuss these subjectivities and label them, isolate them, and package them off. We often use the term *qualia* ^15^ to denote a single “unit” of subjective experience, but this only does a partial job in capturing what that experience IS. Qualia is a nice way of saying that something is a subjective experience, but it cannot evoke that experience, and it cannot tell us what that experience is. You see the color blue. There is a physical instantiation of this experience. A wavelength of light is hitting your retina, and signals are sent to the brain conveying that. The signals will say something about the shade of the blue, maybe its intensity and its brightness—lots of characteristics that can be explained in terms of color physics—but there is also a subjective experience here: the blueness of the blue. The blueness is the qualia in this example, and it is something that cannot be verified. I simply cannot know what it is you are experiencing when presented with “blue.” We can both understand what we see to be blue, but there is no indication that what we see is actually the same. You cannot describe to me this experience of perception in a way that would tell me what it is to see what you consider “blue.” Frank Jackson’s classic “knowledge argument” goes that you could not explain blue to a person raised in a black-and-white room, without showing them—even if they know everything there is to know about the color physics, they still could not understand or imagine the experience of seeing blue until they have actually done so.^16^ Furthermore, Moreland Perkins argues that qualia do not necessarily link to objective causes: a smell does not bear any obvious resemblance to the molecule we inhale. Physical sensations do not necessarily actually take place where we perceive them to; we have referred to sensation.^17^ You could not look at a molecule or a site of pain and know what the experience of that sensation would be to an individual.

All of this is to say that qualia only go so far as a descriptor. It can tell us that there is a sensation, but not what that sensation is. Further still, it does not appear that there actually is any way to communicate that sensation, or, indeed, that it is possible to know what the sensation as perceived by another even is. So, despite my desire for, and faith in, science to explain the brain and explain the mind, to explain what, who, and how we are, it does not seem possible for science to answer this problem—to codify the blueness of blue. Per Nagel, again, “if we acknowledge that a physical theory of mind must account for the subjective character of experience, we must admit that no presently available conception gives us a clue about how this could be done.”^18^ So, there is some aspect of our lived experiences—some aspect of our minds—that cannot be reduced to the physical, at least not in a useful or observable way.

My opponent could jump in here and say that I am endorsing some plain dualism, just as I said I would not do. But I would like to occupy a more subtle ground—presumably, whatever this subjectivity is, it must be in some way a product of its physical basis. Without a physical basis, just as Alex has said, there could be no capacity to perceive qualia, and there could be no experience. This leaves me with an irreconcilable problem—I do not accept the mind as some aetheric force, it must, in some way, exist in our universe, but at the same time, it is not an ontologically simple existence.

## Irreducibility

In some ways, one could argue that this comes close to ideas of an “emergent materialism”; that mind is a novel nonphysical property born of a complex system, and it cannot be reduced to a given physicality in itself.^19^ Mind may be only metaphysically dependent on the brain.^20^ If I were to endorse this, I find myself dangerously close to giving ground to Alex. Even if body is only metaphysically necessary, that may be enough to say that a mind needs one. However, I believe we can introduce sufficient separation.

David Chalmers holds that consciousness, or mind, is entailed by information, and information is an ontologically separate fundamental property of the universe, explaining that:

In physics, it occasionally happens that an entity has to be taken as fundamental. Fundamental entities are not explained in terms of anything simpler. Instead, one takes them as basic, and gives a theory of how they relate to everything else in the world.^21^

This greatly resembles the ways in which we often talk about minds. Chalmers suggests that qualia are informational—the blueness of blue is information of which we are aware, although which we cannot convey to others. Qualia do not follow logically from the physical facts of the brain or body, and so they are what is sometimes referred to by, for example, Derek Parfit as *further facts*.^22^ ^,^^23^ Qualia, then, exist only as information—they are irreducible any further.

Information, or subjective experience, means nothing in and of itself. It is also metaphysically possible that it does not exist without being observed, per the famous “If a tree falls in a forest and no one is around to hear it, does it make a sound?” thought experiment. The process of observation, of receipt of that information, is what I think we refer to as mind. These are cases in which we have a human patient, say in a permanent vegetative state, where the brain architecture exists, but there is no perception, no receipt, or no processing of information, and correspondingly, we see no evidence of a mind. The brain may be the engine, but without informational fuel, what use is that engine?

The corollary of this is that without instantiation of some kind, there is not anything present to receive, to interpret, or to experience the information regarding the blueness of blue. We need a tank in which to pour that fuel. Mind must, therefore, have some physical property—it must be instantiated within the universe. It must exist somewhere in order to accept inputs of information. But it is also nonphysical—it is a gestalt of, for want of a better term, functional informational capacities such as the ability to understand, or to perceive qualia. The mind is inhabiting an odd, quantum space; higher mental processes seem to be somehow phenomenologically different from physical cognitive processes. I am, therefore, forced to admit that my views on this debate may lay closer to property dualism (or at best a nonreductive physicalism) than I perhaps previously thought, with nonphysical mental properties supervening on physical substance. And, this is as close as I will come to saying that my opponent has a point—it appears our minds do need a host or substrate to function, and if you choose to call that a body, then very well. But there is no reason to say that this host need be any specific body.

## Function over Form?

The chick is not the eggshell. But without the shell, the chick could not form. It is conceivably possible to remove the embryo from the shell and place it in another shell, where it will grow. It may now be a slightly different chick, but it will still be a chick. So, it may be with the mind. I do not dispute that modes of instantiation—or to return to the language of our initial question, embodiment—will affect the mind, or rather, that they will affect our subjective experience. It is almost undeniable—if the body is a conduit through which inputs are filtered, the incoming sensations, whatever they are, are going in some way to be colored by the medium through which they are transferred. There are wavelengths of electromagnetism that our *Homo sapiens* eyes cannot receive. As such, looking at a light source does not transfer to us the experience of seeing infrared—although our retinas are being impacted by infrared wavelengths of light. An eye that could see infrared would include that input, and we would presumably subjectively experience it as… something. As I have tried to point out, we cannot know what that experience would be.

If the functionality of the mind is the core of the matter, as I believe, it will presumably proceed to enact these functions—to receive information and to have subjective experiences—no matter its mode of instantiation (or its shell) if we can assume a similar degree of complexity for that embodiment. It may not have these experiences in the same ways, the informational input may be inflected or colored or even expanded by the conduit, but an experience would nonetheless be had. The mind would have performed its role in perceiving that input, and interpreting it as an experience.

The brain-in-a-vat concept^24^ is frequently misused, but it seems apt here to demonstrate this idea of the body as a conduit that does not, in itself, matter. Assuming, for a moment, that such a thing is possible, then the vat—its various, presumably electronic and chemical, inputs would act in the same way as our senses. The mechanism by which these operate does not seem to be significant, but it does not seem unrealistic to suggest that we could, in future, emulate the types of electrochemical activity that enter our brains “naturally.” The mind residing in that vat would still take that activity as an input and experience it subjectively, although that the information would be transferred not through ears or eyes and neurons, but through a chemical soup and wires. So, rather than the mode or medium of ingress, the significant element would be the information contained in that electrochemical activity. The process of perception, of mind, would be unchanged. The mind, then, needs a body, but it could be any sufficiently complex body, and anything that we could conceive of as a body.

## Concluding Remarks

The question “Does a Mind Need a Body” is, essentially, flawed. Each is reliant on the other to constitute themselves. A mind may be metaphysically dependent on a substrate, a host. But a substrate is not a body without a mind—it is just an interchangeable shell. If a mind cannot be reduced to a physicality, then specific embodiment does not matter. It has an effect, and it matters in as much as it does shape the mind, gives it structure, and colors our experiences. But that is a byproduct, not the main event. It seems to me that it is not what a mind *is* but what a mind *does* that matters, and what a mind does is to subjectively experience information, to interpret embodied inputs, and to have purpose in and of itself.

If these capacities are emergent properties that we cannot reduce solely to their underlying biology, properties with some nonphysical element that we cannot objectively measure, about which there is an unsolvable epistemological gap, then this says that the first assumption of our central question is problematic. We do not know, objectively, what a mind is and we cannot know, because we cannot escape our own perspective and subjectivity. We are limited by an inability to be objectively introspective; so, there is a systemic and epistemic barrier to any attempt to understand the basis of our minds.^25^ Just as we cannot know what it is like to be a bat, we can only know what it is like to be a human, and that tells us nothing of the nature of a mind in and of itself, removed from a given host. One method may be to attempt to build a mind for someone or something else, perhaps an artificial general intelligence—but the very inability we have to escape ourselves will likely cause us to create their minds in our own image.

If we cannot know mind beyond its abstract functionality, then we cannot say definitively what it is, and where it resides. If we cannot say where it resides, then we cannot say that that must be in a given body. If we cannot definitely say it must reside within our body, then we cannot definitively say that a mind needs a body. That the mind needs a given body relies wholly on the idea that a mind entirely can be quantified and reduced. Because that argument fails, it cannot be proved, despite how much I may wish it could be, then we cannot in good faith say that a mind *needs* a body in any way that we should care about.

## Alex McKeown’s Rebuttal: Does David *Really* Disagree with Me?

One thing that I am concerned by is the extent to which David is actually *dis*agreeing with me. Perhaps in the process of discussing this, we will tease out what it is he is arguing with me about, but it seems to me there was a lot that he accepted. And on my reading of it, what he accepts should lead him to accept my conclusion rather than his.

For example, David accepts that there needs to be some kind of physical host for mind. He accepts that it has to be *somewhere.* But then, he says it does not have to be any *particular* host and, you know, I suppose I understand what he means by that. But he does not dispute that mind has to be *instantiated* in some way and so he does not dispute the underlying physicalist picture. And once you have accepted that the mind needs to be extended in space *somewhere*, it does not seem to me that there is enormous scope for disagreement.

David also accepts my argument that humans do not necessarily have any privileged account of what counts as a body. There could be many different kinds of bodies, so we are not entitled to point to instances of different kinds of physical instantiations of mind and say that is *definitely not* a body. I flag that I kind of anthropomorphic uncertainty, and David accepts that as well.

So David accepts the physicalist picture and he accepts that we do not have a privileged account of what a body is or is not. Really what he is disagreeing about is whether I am right in saying that there are lots of things that count as bodies, given that this move can be construed as claiming a kind of anthropomorphic certainty to which I am not entitled. However, just because something is different embodied in a way that *we* could not possibly imagine does not mean *they are* not entitled to say that they are embodied.

## David Lawrence’s Rebuttal: Embodiment Does Not Matter

Alex, in his argument, rightly gave a great deal of attention to the idea of embodiment. This is not the first forum in which he and I have taken opposing sides on that subject, and I dare say it will not be the last either.

To rebut, I will return to my characterization of the body—whatever we are understanding that to be—as a “conduit.” I want to make two points about this; the first being to reiterate that the specific body does not seem to me to matter very much. I contended that while embodiment surely does matter in as much as any medium will exert an effect on whatever passes through it, this does not fundamentally change the fact that experiences would still be had, and mind would continue to exist and function. Whatever color or inflection the conduit imparts, the signal still arrives and is still processed, as it were.

## The Banality of Substrate

A hardline, reductive physicalist might liken the brain to a machine, some piece of complex electronics. This is, ironically, a helpful analogy here—electronic circuitry imparts resistance to the electrons flowing through it, but the signals travel. A slightly different alloy used in the circuit would provide a different resistance, and that altered resistance might affect the character of the signal slightly—less strong, say—but it would still travel, and still incite the response at the point of receipt. Providing it is capable of transmitting the signal, the alloy—the conduit—does not matter.

Let us say you were born as an able-bodied, neurotypical person, who suffers some accident that affects your body plan, such as the loss of an appendage. Your embodiment is now altered, and your embodied experience is changed. You would, undoubtedly, experience things differently, even once you were adapted to the physical differences. However—your mind would remain your mind, and you would still experience things by the very same processes that it would have, but for the accident. The changing of your embodied shape or self does not self-evidently change the function or purpose of the mind. If we accept that this function is, in part, to experience subjectivity, and we accept that subjectivity is a, or even the, distinguishing feature of a conscious mind, then we could go further to say that the perception of these subjective experiences is *us* in all the ways which matter.

Here, I would like to invoke John Harris, who discusses the continuation of narrative identity and the possibility of “successive selves” in radical life extension. As he puts it,

Suppose [an individual] has three identities, A, B, and C, descending vertically into the future… A will want to be B, who will remember being A; B will want to become C, who will remember being B but possibly not remember being A.^26^

Although Harris uses this to discuss an extreme life span, it holds true for an ordinary one. We do not generally remember the subjective experience of being a baby, beyond possible flashes of certain physical sensations that made a great impression on us, or maybe broad emotional states. We certainly do not recall qualia—and in many ways, this simply does not matter. We are having experiences as who we are *now*, and who we *were* does not seem to be significant beyond that it led us to the present. Furthermore, our embodiment has changed—no element of our bodies is the same as it was when we were small children, or “A.” This is instrumentally true in a real cellular sense, but also true in as much as we are physically different, larger, a different shape. We have, necessarily, a very different embodied experience of life, and as “C” you likely do not remember—and cannot know—what it is like for a young child to be a young child, just as we cannot know what it is like for a bat to be a bat.^27^

Despite this, we do not consider that that change in embodiment has made us some new person, something significantly different from who we were as “A.” This ought also to be true of the mind. A different shell, or substrate—a different embodiment, here—does not prevent subjective experiences taking place in and of themselves, so what seems to be at stake in our original question—does a mind need a body—is whether or not that body has much to do with the actual function of mind.

One subject of much discussion within transhumanist-leaning literature is the idea of whole-brain emulation, or mind upload. Anders Sandberg and Nick Bostrom suggest that:

The basic idea is to take a particular brain, scan its structure in detail, and construct a software model of it that is so faithful to the original that, when run on appropriate hardware, it will behave in essentially the same way as the original brain.^28^

We can more simply think of this as a perfect copy of the type sometimes considered as a means of digital immortality.^29^ As discussed in my opening argument, a perfect copy seems to require copying the element of subjectivity as well, so let us imagine that our copy achieves this. Once you have sat in the copy machine, the you (you-X) that was copied can get up and walk away (assuming a nondestructive process) and continue living and having subjective experiences. You-X’s mind continues to operate, and you-X’s life is generally unchanged. The copied you, you-Y, branches off. For a single instant, the minds of you-X and you-Y will be identical, until it began to have its own subjective experiences as soon as it came “online.” You-Y would be having different experiences to you-X, by virtue of its inputs being different, despite being processed by the same mental architecture. The inputs would enter through a different conduit, dependent on whatever the copied mind resides in—that is, its body.

The distinction between you-X and you-Y, though, is not because they are instantiated in different ways. It is simply that once these minds are no longer exactly identical, it is not possible for one to know the experience of the other. Their mode of embodiment would affect their experience, just as losing a hand would affect it. However, for both you-X and you-Y, the fundamental fact is that they would still be having subjective experiences, in parallel but in the same manner as the predivergent you. Both you-X and you-Y would remain *you,* although on a new path and unable to know each other’s mind. Their housing does not seem to be significant.

## Malleability

The second point I would like to make is that even if you disagree on the primacy of the function of mind as having experiences, embodiment seems to be malleable. If it is malleable, it cannot be fundamentally significant for the existence of mind.

There is an increasing amount of research into how to manipulate the neurological basis of the hypothetical body schema—our internal “map” of our bodies—into accepting new embodiments.^30^ This is primarily of use in the field of prosthetics, wherein it is desirable to help a user adapt to their prosthesis, consider it “part of their body,” and reduce phenomena such as “phantom limb.” Bioengineers also present a “soft embodiment,”^31^ where neural and cognitive body mechanisms are repurposed to allow the embodiment of nonorganic additions, perhaps even things that are nonbiomimetic. A similar affect can be achieved externally—via application of the body transfer illusion, most recognized as the “rubber hand illusion.” Here, the subject’s organic hand is hidden, and a rubber hand is placed within sight. The hidden organic hand is stroked, but the subject experiences the sensation as though it were in the rubber hand. Perceptual mechanisms can override our knowledge about the material reality of our bodies, and give an illusion of a different embodiment than the strict truth.^32^ If such a simple experiment can induce a new embodied experience—even if only temporary—how fundamental can our true embodiment be to our mind and mental processes?

Furthermore, we induce this in ourselves frequently and without intending to, without the use of illusion. With experience and practice, we frequently describe tools or other objects as being “a part of my body.” A snooker player’s cue becomes an extension of their arm, and they experience the strike of the ball at the tip and not in their hand.^33^ A heavy plant operator need not think about the levers they pull to extend the hydraulic arm, to draw the scoop. The machine moves as though it is an extension of the operator. There are countless other examples—driving being the most common. After an adjustment period, we are simply aware of the bounds of our vehicle, we know the spaces in which it can fit—without needing to get out and measure.

Far from being trite examples, these demonstrate that our embodiment is far from concrete. In all the ways that appear to matter, these nonorganic additions to our bodies function and are experienced as though they *are* part of our bodies. I experience the qualia of the strike on the ball; however, it is mediated, and I go on to have whatever resultant emotion or thought or experience that stems from that. I draw away from the needle threatening “my” rubber hand, because I want to avoid the pain I instinctively think will ensue.^34^ If our embodiment is so malleable, and our experiences continue through these new body parts that are so easily incorporated into our schema, then it does not seem to me that embodiment matters in any significant way.

## The Flawed Language of Mind

The final, brief point I would make perhaps serves both Alex and myself. Our debate has relied on circumlocution, and we have both struggled to articulate entirely accurately what we think mind to be. This is borne out across the literature of the mind–body problem and more widely in philosophy of mind—it is extremely difficult to effectively discuss something which is, by its nature, entirely nebulous and unknown. Furthermore, we are limited by the language we have available to us. All the terminology we use—“mind,” “consciousness,” and even “body”—can be understood in myriad ways. We invoke possessive terms constantly, and in so doing necessarily suggest that “my” mind is “me,” when this view is not shared—for instance, by my learned opponent. We cannot describe qualia, because we utterly lack the words for it, in any language. We analogize—and I have done so extensively here—because we are trying to describe an invisible and quite possible nonphysical process, but the analogies themselves are limited to things that we *can* observe, things that, therefore, fundamentally cannot entirely represent such a process.

Until we can solve some of these issues—until we can agree some definitions, some limits—it does not seem likely that this is a debate we can conclude one way or the other. I maintain, then, that even if my arguments in these statements fail in themselves, there must remain a reasonable doubt that mind “needs” body.

## Alex McKeown: David Lawrence’s Best Argument

The strongest argument David makes relates to the use of the definite article when talking about mind, given we do not we do not know what “a” mind is and that it is probably better understood as a “functional Gestalt.” A key difference between mind and body is that the body, which in humans and other animals includes the brain, can be seen and observed by empirical investigation, whereas even though processes of mind are physically instantiated, you are not going to open up a brain and find “a mind.” Rather, “a” mind is a shorthand for describing a collection of functions that are characteristic of the kinds of beings that we are.^35^

This of course admits a degree of uncertainty about what mind is and is not, however, so David’s skepticism about this is reasonable and there is a risk that I am in fact over-anthropomorphizing the picture. So, to me, that is probably David’s strongest argument, because there is some legitimate skepticism about what “a mind” is and where it resides. There is also the general skepticism about knowledge of other minds because of the radical uncertainty about what other people’s subjective experience is like.

As an aside, of course, if one were a panpsychist—we all know that the panpsychist debate has been raging recently and let’s not get into that here— one could just say, “well, the mental is an intrinsic feature of the physical, because this sidesteps the hard problem of emergence”. Not all experience will be as sophisticated as our mental events, and matter in general might just have an unimaginably primitive form of experience; that is, one that is not self-aware or self-referential and so on. Nevertheless, if you buy the conclusion that matter is experiencing, because this is a more parsimonious explanation for the existence of mind than the dualist picture in which mind and body are separate, then you might have some sympathy with the view that there is necessarily no non-physically instantiated mind.

Having said this, and to finish off, I think David articulated his strongest point well at the end there, which is that in spite of these arguments it is possible to have reasonable doubt that mind and body are inseparable. There really does *seem* to be something *different* about mental experiences from physical ones, we experience those things differently, and this phenomenological aspect is a challenge that might instill reasonable doubt as to whether, in fact, you cannot have one without the other.

## David Lawrence: Alex McKeown’s Best Argument

For all my contention that the mode of embodiment does not matter, I have to accept—and I was forced to admit during my own arguments—that I find it nearly impossible to deny the necessity of physicality, even if that necessity is purely instrumental. All phenomena must be grounded in the physical, in some way even those we cannot easily reduce to it. There is no aetheric substance experiencing subjectivity—the mind, whatever it proves to be, must be in some way instantiated. It must be subject to the fundamental laws of physics, even if it may be that we do not entirely understand how as yet. If subjectivity is information, and if the information can be considered a fundamental component of physics—as Chalmers might have it—then perhaps subjectivity, too, has a rational scientific explanation to be discovered. Physicality does not have to mean we can touch something, merely that it be subject to physics, and materialism such as that which Alex relies on merely requires that there is matter and material interaction in the process of mind. In this regard, I cannot deny him.

This problem returns us to one of the flaws of our central debate topic—what we understand to count as a body. It is a reasonable argument to make to say that whatever host, substrate, or conduit mind resides in could be called its body. If so, then that is impossible to repudiate—even if it makes “body” so vague a concept as to be useless.

## Notes

1.

Strawson G . Real naturalism. Proceedings and Addresses of the American Philosophical Association 2012;86(2):125–54; and Strawson G . Real intentionality. Phenomenology and the Cognitive Sciences 2004;3(3):287–313 .

2.

Chrisley R . Embodied artificial intelligence. Artificial Intelligence 2003;149(1):131–50, at 132.

3.

Vernon D , Furlong D . Philosophical foundations of AI. In: Lungarella M , Lida F , Bongard J , Pfiefer R , eds. 50 Years of Artificial Intelligence. Berlin and Heidelberg: Springer; 2007:53–63, at 60.

4. Ziemke T. Disentangling notions of embodiment. *Workshop on Developmental Embodied Cognition* 2001;83–8.

5.

Duffy B , Joue G . Intelligent robots: The question of embodiment. In: Proceedings of the Brain–Machine Workshop 2000;1–8, at 3; and Prem E . Epistemological aspects of embodied artificial intelligence. Cybernetic Systems 1997;28(5):3–9 .

6.

Mataric MJ . Studying the role of embodiment in cognition. Cybernetic Systems 1997;28(6):457–70, at 460.

7.

Kim J . Mind–body problem. In: Honderich T , ed. Oxford Companion to Philosophy. Oxford: Oxford University Press; 1995:579–80.

8.

Crane T , Patterson S . Introduction. In: Crane T , Patterson S , Eds. History of the Mind–Body Problem. New York: Routledge; 2001:1–2 .

9.

Stack GJ . Materialism. In: Craig E , ed. Routledge Encyclopedia of Philosophy: Luther to Nifo. London: Routledge; 1998;171–2.

10. Wolfendale P. Interview: Artificial bodies and the promise of abstraction*. The Philosopher* 2020;**108**(3); available at https://www.thephilosopher1923.org/interview-wolfendale (last accessed 7 Mar 2021).

11. For example, by functional magnetic resonance imaging: Huettel SA, Song AW, McCarthy G. *Functional Magnetic Resonance Imaging.* 2nd ed. Sunderland, MA: Sinauer; 2009; or functional ultrasound: Mace E, Montaldo G, Cohen I, Fink, M, Tanter, M. Functional ultrasound imaging of the brain: Theory and basic principles*. IEEE Transactions on Ultrasonics, Ferroelectrics and Frequency Control* 2003;60(3):492–506.

12.

Nagel T . What is it like to be a bat? The Philosophical Review 1974;83(4):435–50.

13.

Dennett DC . Consciousness Explained. Boston: Little, Brown and Company; 1991, at 442.

14.

Chalmers D . The Conscious Mind: In Search of a Fundamental Theory. Oxford: Oxford University Press; 1997 .

15. Tye M. Qualia. In: Zalta EN, ed. *The Stanford Encyclopedia of Philosophy.* Stanford University; 2018; available at https://plato.stanford.edu/entries/qualia (last accessed 7 Mar 2021).

16.

Jackson F . Epiphenomenal Qualia. The Philosophical Quarterly 1982;32(127):127–36.

17.

Perkins M . Sensing the World, Indianapolis: Hackett; 1982, at 31.

18. See note 12, Nagel 1974.

19.

Martinich AP , Sosa ED . *A Companion to Analytic Philosophy.* Malden, MA: Blackwell Publishing; 2008, at 65.

20.

Sprevak M , Kallestrup J . New Waves in Philosophy of Mind. London: Palgrave Macmillan; 2014, at 158.

21.

Chalmers D . Facing up to the problem of consciousness. Journal of Consciousness Studies 1995;2(3):200–19.

22.

Parfit D . Reasons and Persons. Oxford: Oxford University Press; 1986, at 309–10.

23. See note14, Chalmers 1997, at 84–7.

24.

Harman G . Thought. Princeton: Princeton University Press; 1973, at 5.

25. Thanks to Dr Joshua Shepherd for making this point in discussion.

26.

Harris J . Enhancing Evolution. Princeton; Princeton University Press; 2007, at 65.

27. See note 12, Nagel 1974.

28. For instance: Sandberg A, Bostrom N. Whole brain emulation: A roadmap. *Technical Report #2008-3.* Future of Humanity Institute, Oxford University; available at www.fhi.ox.ac.uk/reports/2008–3.pdf (last accessed 7 Mar 2021).

29.

Martin GM . Brief proposal on immortality: An interim solution. Perspectives in Biology and Medicine 1971;14(2):339–40554625810.1353/pbm.1971.0015.

30. See, for instance, Segura Meraz N, Sobajima M, Aoyama T, Hasegawa Y*.* Modification of body schema by use of extra robotic thumb. *Robomech Journal* 2018;5(3):1–8; and Bekrater-Bodmann R. Perceptual correlates of successful body–prosthesis interaction in lower limb amputees: Psychometric characterisation and development of the Prosthesis Embodiment Scale. *Scientific Reports* 2020;10(1):14203.

31.

Makin TR , de Vignemont F, Micera S. Soft embodiment for engineering artificial limbs. Trends in Cognitive Sciences 2020;24(12):965–83312972110.1016/j.tics.2020.09.008.

32. Kammers MP, van der Ham IJ, Dijkerman HC. Dissociating body representations in healthy individuals: Differential effects of a kinaesthetic illusion on perception and action. *Neuropsychologia* 2006;44(12):2430–6.

33. Wolfendale P. Interview: Artificial bodies and the promise of abstraction*. The Philosopher* 2020;**108**(3); available at https://www.thephilosopher1923.org/interview-wolfendale (last accessed 7 Mar 2021).

34. Carlson NR. *Physiology of Behavior.* 11th ed. New York: Allyn & Bacon; 2010.

35.

Smit H , Hacker PMS . Seven misconceptions about the mereological fallacy: A compilation for the perplexed. Erkenntnis 2014;79(5):1077–97; and Hacker PMS . The relevance of Wittgenstein’s philosophy of psychology to the psychological sciences. In: Proceedings of the Leipzig Conference on Wittgenstein and Science 2007;1–23 *.*

